# Melatonin binds with high affinity and specificity to beta‐amyloid: LC‐MS provides insight into Alzheimer’s disease treatment

**DOI:** 10.1002/2211-5463.13279

**Published:** 2021-09-01

**Authors:** Yaqian Dai, Liduo Peng, Yajing Liu, Yuanhong Xu, Jinping Qiao

**Affiliations:** ^1^ Department of Clinical Laboratory The First Affiliated Hospital of Anhui Medical University Hefei China; ^2^ Department of Clinical Laboratory Anqing Municipal Hospital Anqing China; ^3^ Department of Obstetrics and Gynecology The First Affiliated Hospital of Anhui Medical University Hefei China; ^4^ NHC Key Laboratory of Study on Abnormal Gametes and Reproductive Tract (Anhui Medical University) Hefei China

**Keywords:** Alzheimer's disease, beta‐amyloid, melatonin, receptor binding assay, liquid chromatography–mass spectrometry

## Abstract

To study the potential relationship between melatonin and beta‐amyloid (Abeta), we established a liquid chromatography–mass spectrometry (LC‐MS) method to quantitatively analyze melatonin, deuterated isotopes (melatonin‐D4), and internal standard 6‐iodo‐2‐(4'‐dimethylamino‐) phenyl‐imidazo(1,2) pyridine (IMPY) under positive (+) mode. The gradient elution was set to 6 min, and the corresponding peak time of melatonin and its isotope melatonin‐D4 was 3.14 min, while the peak time for the internal standard IMPY was 3.24 min. Next, we established and optimized the molecule receptor saturation binding assay based on LC‐MS to determine the melatonin affinity for beta‐amyloid (Abeta). Melatonin showed a high and specific binding for Abeta. The corresponding equilibrium dissociation constant (*K*d) of melatonin with Abeta 1‐40 and Abeta 1‐42 was 814.37 ± 36.62 and 628.33 ± 13.57 nmol·L^−1^; besides, the *K*d of melatonin with mixed plaques (1‐40 and 1‐42) was 461.13 ± 45.37 nmol·L^−1^. The results may suggest the potential mechanism of action of MT on Abeta and provide a theoretical basis for the improvement of MT treatment of Alzheimer's disease.

## Graphical abstract

Our study is the first to show the direct binding of melatonin with beta‐amyloid, suggests the potential mechanism of action of MT on beta‐amyloid, and provides a theoretical basis for the improvement of MT treatment of AD.

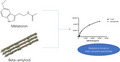

**Abbreviations**
AD: alzheimer's disease
MT: melatonin
MT‐D4: melatonin‐D4
Abeta: beta‐amyloid
LC‐MS: liquid chromatography–mass spectrometry
*K*d: equilibrium dissociation constant
SPs: senile plaques
NFTs: neurofibrillary tangles

Alzheimer's disease (AD), characterized by cognitive dysfunction and memory loss, is a chronic progressive neurodegenerative disease. Development of brain senile plaques (SPs) from the deposition of beta‐amyloid (Abeta) protein, mainly the Abeta 1‐40 and Abeta 1‐42, is one of the key pathological features of AD [1, 2]. This usually leads to neurofibrillary tangles, cell loss, vascular damage, and dementia [3]. The administration of melatonin (MT, N‐acetyl‐5‐methoxytryptamine), a neurohormone with antiaging, antideterioration, and antioxidant properties, is considered as a potential treatment of senile dementia [4]. MT, produced by the pineal gland, affects circadian rhythm and seasonal reproduction. In addition, it is a highly efficient local regulator in all tissues [5, 6, 7]. MT treatment in AD animal models reduced the accumulation of Abeta and hyperphosphorylated tau protein, improved the neuroplasticity and neuronal survival ability, slowed down cognition and memory disorders, and reduced anxiety and depression behaviors, suggesting the antiaging neuroprotective effect of MT [8, 9, 10]. Meanwhile, clinical studies showed that MT expression was reduced in the cerebrospinal fluid (CSF) of aging and AD patients. External administration of MT not only inhibits the Abeta deposition but also prevents the formation of amyloid fibrils through structural interaction, which improves sleep quality and slows cognitive impairment in AD patients [11]. However, the mechanism of action of MT is not yet fully clear; specifically, whether MT can bind to Abeta is uncertain. This study established and improved the molecule receptor binding method based on the radioligand receptor binding assay [12], based on liquid chromatography–mass spectrometry (LC‐MS), to measure the MT affinity for Abeta. Our results provide the theoretical basis for MT action on Abeta.

## Materials and methods

### Materials

#### Reagents

Melatonin (C13H16N2O2, 232.28 g·mol^−1^) and melatonin‐D4 (C13H12N2O2D4, 236.28 g·mol^−1^) were purchased from Sigma (USA). Abeta 1‐40 and Abeta 1‐42 proteins were purchased from RiboBio (China). Methanol and bovine serum albumin (BSA) were purchased from TEDIA (USA) and Bioengineering Co., Ltd. (China), respectively.

#### Instruments

AB Sciex Mass Spectrometer (API 3200, USA), Shimadzu Nexera X2 UPLC System (Japan), Q Exactive Plus Hybrid Quadrupole Orbitrap Mass Spectrometer (Thermo Scientific, San Jose, USA) with a heat electrospray ionization (HESI), ZT‐II Cell Collector (Satellite Medical Equipment Manufacturing Co., Ltd., Shaoxing City), DSH‐300A Water Bath Constant Temperature Oscillator (Peiying Experimental Equipment Co., Ltd., Suzhou City), BE‐9010 Microplate Constant Temperature Oscillator (Qilin Bell Instrument Manufacturing Co., Ltd., Haimen City), and 5810R desktop centrifuge (Eppendorf, Germany) were used in this study.

### Methods

#### Establishment of LC‐MS method for MT, MT‐D4, and IMPY

Based on MT MW (molecular weight), MT‐D4 and IMPY solutions (1 × 10^−2^ mol·L^−1^) were prepared in dimethyl sulfoxide (DMSO) and then diluted with 80% methanol (Milli‐Q ultrapure water: methanol = 1 : 4) to obtain the working solutions (1 × 10^−7^ mol·L^−1^). The MS parameters were set following the conventional method in the MRM (multiple reaction monitoring) mode, and the system was optimized for respective ion pairs using the declustering potential (DP), entrance potential (EP), collision energy (CE), collision cell exit potential (CXP), temperature (TEM), ionization voltage (IS), curtain gas (CUR), GS1, GS2, and collision gas (CAD). MS was connected to the liquid chromatography (LC) system with the mobile phase Milli‐Q ultrapure water containing 0.1% formic acid (pump A), and methanol containing 0.1% formic acid (pump B). The LC conditions were optimized based on MS methods in the LC‐MS mode with repeated injection. The flow rate was 0.4/min, and the column temperature was 40 ℃. The injection volume was 20 μL, and the gradient elution was set to 6 min. The optimized MS and LC conditions were used to analyze MT, MT‐D4, and IMPY.

#### Establishment of the quantitative LC‐MS assay for MT, MT‐D4, and IMPY

MT stock solution (1 × 10^−2^ mol·L^−1^) was diluted with 80% methanol to obtain 5 distinct standard solutions (1 × 10^−6^ to 1 × 10^−10^ mol·L^−1^). Likewise, the internal standard solution (IMPY, 1 × 10^−7^ mol·L^−1^) was prepared with 80% methanol. 450 μL of MT standard solution was added to 50 μL of internal standard solution (IMPY, 1 × 10^−7^ mol·L^−1^), and mixed by vortexing to obtain 5 distinct concentration standard solutions. Standard solutions, from low to high concentrations, were detected using the above‐described LC‐MS method to quantitate MT, MT‐D4, and IMPY following the method guide. IMPY was designated as the internal standard, and MT was selected as the quantitative ion. The theoretical concentration was calculated by fitting a standard curve after manual or automatic integration, and the correlation coefficient (R‐value) was determined.

#### Saturation binding experiment

The experimental method is based on the radioactive receptor saturation binding method but without using any radioactive ligands [13]. Firstly, Abeta 1‐40 and Abeta 1‐42 were prepared with ammonia water (0.02%). Then, PBS (0.04 mol·L^−1^) was added (1 : 9) to obtain the final concentration of 5 × 10^−5^ mol·L^−1^. This was incubated at 37 ℃ for 96 h on a vertical mixer and then further diluted 5 times with PBS (0.01 mol·L^−1^) to obtain the final Abeta solution (1 × 10^−5^ mol·L^−1^). The reaction system was divided into 9 tubes as the total binding tubes (TB) and 3 tubes as the nonspecific binding tubes (NSB), using 12 mm × 75 mm glass tubes (Fisher Company, USA). The sequence of sample addition to the total combination tube was as follows: 1400 μL 0.1% PBS/BSA (obtained by diluting BSA with 0.01 mol·L^−1^ PBS); 200 μL MT (3 × 10^−5^ to 1.1719 × 10^−7^ mol·L^−1^, diluted twice sequentially); and 400 μL Abeta solution (1 × 10^−5^ mol·L^−1^). To the nonspecific binding tubes, 200 μL of the competitive ligand MT‐D4 (3 × 10^−5^ mol·L^−1^) was added and the final volume was fixed to 2 mL. Tubes were sealed with Parafilm, vortexed for 5 min, then incubated in water for 2 h (37 ℃, 200 r·min^−1^). A ZT‐II‐type cell collector equipped with two pieces of Whatman GF/B filters, which were soaked in 0.5% polyethyleneimine (PEI) for 2 h at 4 ℃ in advance, was used to separate the Abeta fiber‐bound MT. 10% ethanol was used for sequential elution to remove free MT, which was reconstituted with 1350 μL methanol on a microplate thermostatic shaker (37 ℃, 1000 r·min^−1^) for 30 min. Next, 150 μL internal standard solution (IMPY, 1 × 10^−7^ mol·L^−1^) was added with vortexing. 1000 μL of supernatant was taken out into a 96‐deep‐well‐plate, centrifuged at 15 000 r·min^−1^ for 5 min, and then dried with nitrogen at 40 °C. This was reconstituted with 80% methanol (800 rpm·min^−1^, 30 min). The LC‐MS method established in Section ‘3.2.1’ for MT, MT‐D4, and IMPY was used for analysis. After the detection, the quantitative method established in Section ‘3.2.2’ was used for data integration and quantification. The results were analyzed by graphpad Prism 8.0 to obtain the equilibrium binding constant (*K*d value). Three independent experiments were performed, and each experimental group included two replicates. The final values denote *K*d ± standard error from three experiments.

## Results and discussion

### Results

#### Establishment of mass spectrometry (MS) method

MS identification methods for MT, melatonin‐D4 (MT‐D4), and IMPY (6‐iodo‐2‐(4'‐dimethylamino‐) phenyl‐imidazo (1,2) pyridine) in the positive (+) mode were successfully established. Parent and corresponding product ions were analyzed based on the standard substance. Declustering potential (DP), entrance potential (EP), collision energy (CE), and collision cell exit potential (CXP) were optimized to improve analysis by increasing the response value of product ion by 2–3 times than that of the parent ion. The final MS conditions were as follows: curtain gas (CUR), 35 psi; collision gas (CAD), 10 psi; ionspray voltage (IS), 4500 V; heated capillary temperature (TEM), 550 ℃; and GS1 and GS2, 40 psi (both). The optimized parameters for the respective compound are shown in Table 1.

**Table 1:** Optimized MS/MS parameters for melatonin, melatonin‐D4, and IMPY.

Compound	m/z	DP	EP	CE	CXP	CUR	CAD	IS	TEM	GS1	GS2
Melatonin	233.1/174.1	40	7	20	4	35	10	4500	450	40	40
	233.1/159.1	44	8	40	5	35	10	4500	450	40	40
Melatonin‐D4	237.2/178.2	48	8	25	3	35	10	4500	450	40	40
	237.2/163.3	45	6	30	3	35	10	4500	450	40	40
IMPY	364.1/236.3	60	10	48	3	35	10	4500	450	40	40
	364.1/348.2	70	7	49	7	35	10	4500	450	40	40
	364.1/193.3	80	10	67	3	35	10	4500	450	40	40

#### Establishment of liquid chromatography (LC) method

The LC device was connected to the MS, and the liquid chromatography elution conditions were established successfully. The gradient elution time was set to 6 min with settings as follows (Table 2): pump A, Milli‐Q water with 0.1% formic acid; pump B, methanol with 0.1% formic acid; gradient setup, 0.01–1.0 min (1% B), 1.0–2.0 min (1–98% B), 2.0–3.5 min (98% B), 3.5–4.5 min (98–1% B), and 4.5–6.0 min (1% B); flow rate, 0.4 mL·min^−1^; and sample injection, (single) 20 μL. The peak time of MT and its isotope internal standard MT‐D4 was 3.14 min, while the peak time of IMPY was 3.24 min.

**Table 2:** Elution gradient conditions of liquid chromatography.

Time(min)	Module	Pump B^a^		Pump A^a^	
		Event	Parameter	Event	Parameter
0.01	Pumps	Pumps B Conc.	1	Pumps A Conc.	99
1.00	Pumps	Pumps B Conc.	1	Pumps A Conc.	99
2.00	Pumps	Pumps B Conc.	98	Pumps A Conc.	2
3.50	Pumps	Pumps B Conc.	98	Pumps A Conc.	2
4.50	Pumps	Pumps B Conc.	1	Pumps A Conc.	99
6.00	Controller	Stop		Stop	

^a^ Pump A: Milli‐Q water containing 0.1% formic acid; Pump B: methanol containing 0.1% formic acid.

#### Saturation binding assay: determination of dissociation constant (Kd)

**Fig. 1:**
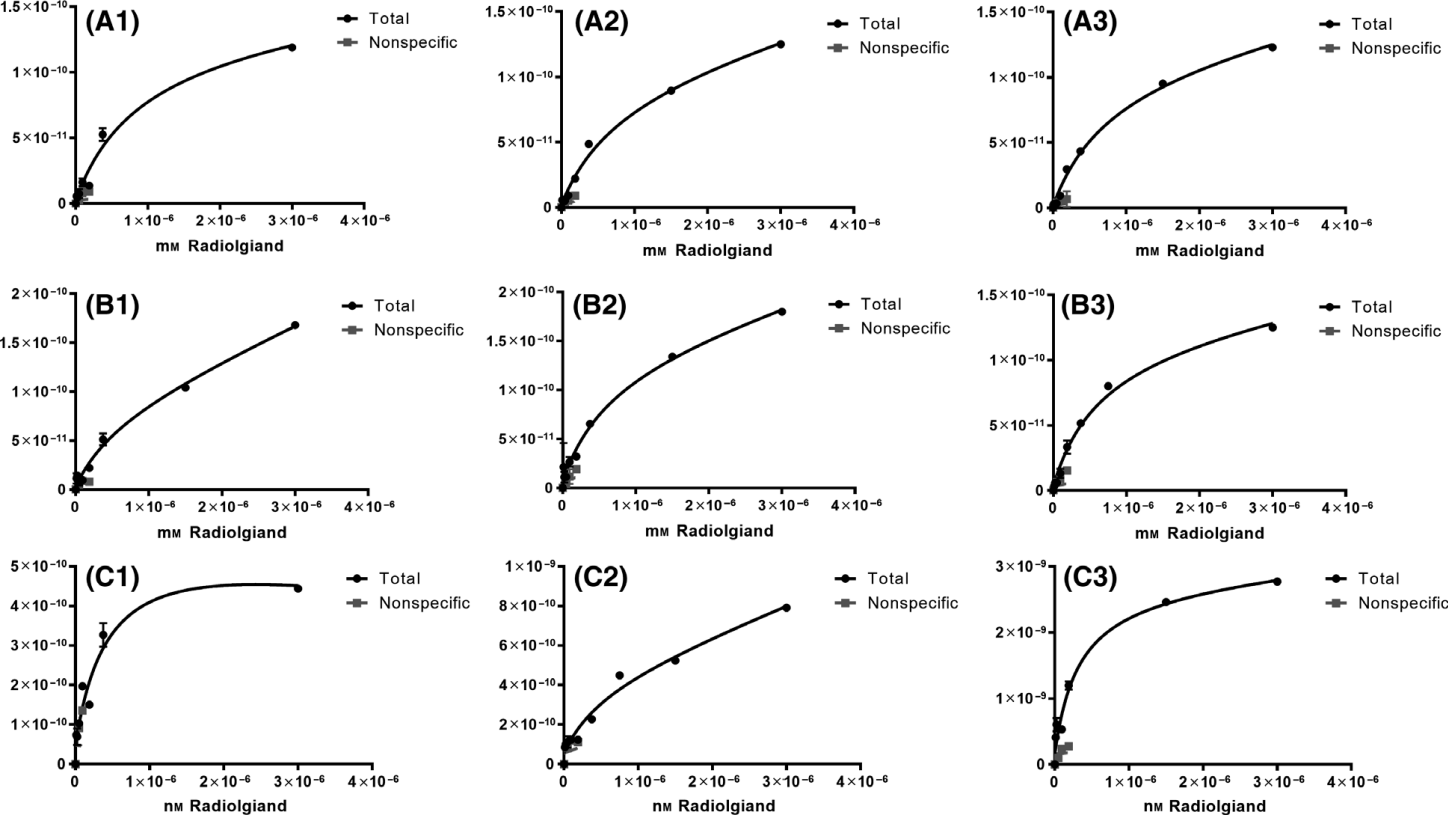
Saturation binding curves. A1, A2, and A3 are the saturated binding curves for melatonin with Abeta 1‐40 protein; B1, B2, and B3 are the saturated binding curves for melatonin with Abeta 1‐42 protein; and C1, C2, and C3 are the saturated binding curves for melatonin with Abeta mix. ^a^Three independent experiments were performed (*n* = 3), each experimental group included two replicates, and the error bars indicate DV (difference value).

MT could specifically bind to Abeta 1‐40 and Abeta 1‐42, and the respective Kd values were 814.37 ± 36.62 and 628.33 ± 13.57 nmol·L^−1^ (Table 3); the Kd of melatonin with mixed plaques (1‐40 and 1‐42) was 461.13 ± 45.37 nmol·L^−1^. Three independent experiments were performed, while each experiment had two replicates. The Kd and the saturation binding curves of MT with Abeta fibers were analyzed by graphpad Prism 8.0. The final values denote the Kd ± standard error from three experiments; the CV (coefficient of variation) values of the three experiments were within 15%. The three sets of saturated binding curves, shown in Fig. 1, corresponded to the respective Kd values in Table 3.

**Table 3:** Equilibrium binding constants (Kd) of melatonin with Abeta fibers (nmol·L^−1^).

	Abeta1‐40	Abeta1‐42	Abeta mix^b^
	Kd ± SEM (nm)	Kd ± SEM (nm)	Kd ± SEM (nm)
Kd (Experiment 1)	887.60	603.40	466.5
Kd (Experiment 2)	778.10	650.10	536.9
Kd (Experiment 3)	777.40	631.50	380
Mean	814.37	628.33	461.13
Standard error	36.62	13.57	45.37
CV^a^	4.5%	2.2%	9.8%

^a^ CV, mean/ standard error.

^b^ Abeta mix means the mixed plaques (1‐40 and 1‐42), and the final Abeta solution is 5 × 10^−6^ mol·L^−1^.

### Discussion

AD, affecting ˜ 50 million people worldwide, is the most common cause of dementia. Regrettably, the number of AD patients is expected to be doubled every 20 years, causing serious economic and social burdens [14]. SPs formed by Abeta deposition and neurofibrillary tangles (NFTs) formed by highly phosphorylated tau protein are the two main pathological features of AD [15]. In addition, AD patients usually suffer from sleep disturbance, and the lack of sleep increases with the progression of the disease. During the early events of Abeta deposition in the brain, patients do not show clear signs of cognitive impairment; however, the MT level in the CSF starts reducing, which is positively correlated to the disease progression [16]. The reason for the decline of MT level is yet unknown.

MT is a circadian rhythm regulating hormone and promotes neuroprotection through receptor‐dependent and receptor‐independent mechanisms. The MT expression starts increasing from birth and reaches a peak around puberty, followed by a gradual decrease with aging [17, 18]. Studies showed that MT functions via MT1 and MT2 receptors, activating various signal transduction pathways to regulate key proteins, such as ADAM10, BACE1, and GSK‐3, which in turn controls Abeta synthesis by clearing the deposits [19, 20].

The LC‐MS, which can identify and quantify target analytes with high accuracy and resolution, offers the advantage of high separation efficiency of LC for heat‐sensitive and high boiling point compounds with the strong component identification ability of MS [21]. In this study, an LC‐MS platform was used to determine the MT affinity for Abeta. We established and optimized a nonradioactive small molecule receptor binding assay based on the radioligand binding assay (RBA); it could reduce the risk and difficulty of the experiment greatly, and reduce the work intensity of the experimenter, which could be completed in an ordinary laboratory. Target substances bound to Abeta were intercepted by using the two pieces of Whatman GF/B filter membranes, and then, the large volumes of samples were dried with nitrogen and finally redissolved in a small volume. This improved the detection range with higher accuracy and efficiency.

Here, we established and optimized a receptor binding assay for nonradioactive small molecules based on LC‐MS. To validate the method, we determined the affinity of IMPY with Abeta fibers using this method and obtained the IMPY *K*d values, 3.80 ± 0.39 and 18.38 ± 1.11 nmol·L^−1^ for Abeta1‐40 and Abeta1‐42 fibers, respectively. Notably, this is consistent with the previously reported value (*K*d = (5.3 ± 1.0) nmol·L^−1^); however, the previous study did not distinguish between Abeta1‐40 fibers and Abeta1‐42 fibers [22]. Furthermore, MT affinity (*K*d) for Abeta 1‐40 and Abeta 1‐42 was found to be 814.37 ± 36.62 and 628.33 ± 13.57 nmol·L^−1^, respectively, showing a strong binding; besides, the *K*d of melatonin with mixed plaques (1‐40 and 1‐42) was 461.13 ± 45.37 nmol·L^−1^, this is more in line with the Abeta deposition in the patient’s brain. We believe that our study is the first to show the direct binding of MT with Abeta fibers.

Also, our results suggest the potential mechanism of action of MT on Abeta fibers. Past studies show that the Abeta fiber content of CSF increases gradually with the progression of AD, while the MT decreases, correspondingly. Therefore, one can speculate that the phenomenon could be related to that the increased binding of MT to Abeta fibers decreases the MT concentration of CSF in AD patients. MT is a highly effective endogenous hormone that is widely distributed in all tissues. With strong lipophilicity, MT easily reaches the brain to exert neuroprotective functions. *In vitro* studies showed that MT treatment reduces the accumulation of Abeta fibers in the brain and deaccelerates sleep and cognitive impairment. Moreover, the specific affinity of MT for Abeta can explain its depolymerization effect on Abeta fibers, providing new insights into its mechanism of action. This study provides a theoretical basis for the improvement of MT treatment of AD.

In addition, we achieved the determination of *K*d by using Whatman GF/B filters to withhold the compound that bound to Abeta protein based on the molecular weight of Abeta protein, so it not only was suitable for the study of melatonin, but also provided an efficient and feasible screening system for Abeta‐based probes for early diagnosis, which will promote the development of Abeta molecular imaging probes certainly, especially nonradioactive probes such as MRI and CT. It is also worth noting that the appropriate filters can be selected according to the molecular weight of the protein, so this method can also be applied to the study of the affinity determination between other proteins and ligands.

## Conflict of interest

The authors report no conflicts of interest for this work.

## Author contribution

Jinping Qiao and Yuanhong Xu conceived and designed the project. Yaqian Dai and Liduo Peng acquired the data. Yaqian Dai and Yajing Liu analyzed and interpreted the data. Yaqian Dai and Jinping Qiao wrote the paper.

## Data Availability

The data that support the findings of this study are available from the corresponding author (jpqiao@ahmu.edu.cn) upon reasonable request.
